# Controlled Morphing of Microbubbles to Beaded Nanofibers via Electrically Forced Thin Film Stretching

**DOI:** 10.3390/polym9070265

**Published:** 2017-07-03

**Authors:** Zhi-Cheng Yao, Qiantailang Yuan, Zeeshan Ahmad, Jie Huang, Jing-Song Li, Ming-Wei Chang

**Affiliations:** 1 Key Laboratory for Biomedical Engineering of Education, Ministry of China, Hangzhou 310027, China; zhicheng_yao@zju.edu.cn (Z.-C.Y.); yqtl@zju.edu.cn (Q.Y.); ljs@zju.edu.cn (J.-S.L.); 2 Zhejiang Provincial Key Laboratory of Cardio-Cerebral Vascular Detection Technology and Medicinal Effectiveness Appraisal, Zhejiang University, Hangzhou 310027, China; 3 Leicester School of Pharmacy, De Montfort University, The Gateway, Leicester LE1 9BH, UK; zahmad@dmu.ac.uk; 4 Department of Mechanical Engineering, University College London, London WC1E 7JE, UK; jie.huang@ucl.ac.uk

**Keywords:** bubble, beaded nanofibers, electrospinning, polyvinyl alcohol

## Abstract

Topography and microstructure engineering are rapidly evolving areas of importance for biomedical and pharmaceutical remits. Here, PVA (Polyvinyl alcohol) microbubbles (diameter range ~126 to 414 μm) were used to fabricate beaded (beads-on) nanofibers using an electrohydrodynamic atomization (EHDA) technique. Mean fiber diameter, inter-bead distance, and aspect ratio (AR) were investigated by regulating EHDA process parameters. PVA fibers (diameter range ~233 to 737 nm) were obtained possessing bead ARs in the range of ~10 to 56%. AR was used to modulate hydrophilicity and active release.

## 1. Introduction

To date, near uniform fibers (as opposed to those exhibiting broader irregularities) have been preferred for pharmaceutical and biomaterial applications such as drug delivery [1], wound healing [2], and food preservation [3]. The electrospinning (ES) process, which is functional in an EHDA experimental setup, utilizes an electrostatic field for the production of continuous fibers, and has attracted significant attention due to numerous engineering advantages over other well-established fabrication methods (e.g., ambient environment operation). Furthermore, a greater preference for fibrous materials is emerging, which provides options to tailor physical and mechanical properties with greater ease. The porous nature of filamentous structures (e.g., films, membranes) yields additional benefit from inherently high aspect ratios (ARs) [4]. During ES/EHDA processes, surface morphology and diameter are modulated through process parameters (e.g., polymer concentration, applied voltage, solution flow rate, and collector distance) [5]. In recent times, fiber structure diversification through ES has been shown by the engineering of Janus, twisted, and core-shell structures [6,7,8]. In addition to these structures, beads-on (or beaded) fibers are also emerging as valuable architectures, even though the uniformity of such materials is significantly different to their perfectly electrospun counterparts [9]. Beaded fibers are commonly prepared (when using ES) by deploying solutions possessing low polymer concentrations [10]. While subsequent structures appear irregular, the aggregation of impregnated chemicals (such as drugs) within ‘beads’ have shown to modulate their release behavior from the resulting non-woven materials [11,12]. In addition, when compared to equivalent non-beaded structures, beaded fibers exhibit a greater contact area. This is an ideal property for biological scaffolds, which are known to improve cell growth [13,14]. However, with the main emphasis to date on smooth regular fibers, the impact of the process parameters on beaded fiber optimization remains limited. The synthesis of such irregular morphologies has often been shown as part of the process medium validation during ‘pristine’ ES. Another family of microstructures currently being explored for numerous advances in biomedical engineering is microbubbles [15]. In general, such structures are described as a gaseous material encapsulated by a thin film, which in many cases is a polymeric material [16,17].

Herein, we demonstrate a novel fabrication route for the controlled engineering of beaded fibers using microbubbles. Polyvinyl alcohol (PVA), a biocompatible synthetic polymer, was used to prepare a series of microbubble suspensions (using deionized water as the vehicle) with varying polymer loading volumes [18]. Suspensions were subsequently subjected to the EHDA process leading to continuous beaded fiber production. Fiber property control (diameter, inter-bead distance, and AR) and the resulting beaded fibrous film surface hydrophilicity were investigated via conventional EHDA processing parameters. Finally, a variety of drug (Tetracycline (TE)) hosting beaded fiber matrices was prepared and the drug release was controlled through tailored fiber properties.

## 2. Materials and Methods

### 2.1. Materials

Polyvinyl alcohol (PVA, Mw 7.48 × 10^4^ g/mol, degree hydrolyzed 88%) was purchased from Sigma-Aldrich Ltd., St. Louis, MO, USA. Tetracycline hydrochloride (TE-HCL) was obtained from Amresco, Solon, OH, USA. Phosphate buffer solution (PBS, pH 7.4) was obtained from Sinopharm Chemical Reagent Co., Ltd (Shanghai, China). Deionized (DI) water with a resistivity of 18.2 MΩ cm at 25 °C was produced using a Millipore Milli-Q Reference ultra-pure water purifier (Merck Millipore, Billerica, MA, USA). All chemicals used were analytical grade without additional purification.

### 2.2. Methods

#### 2.2.1. Beads-On Nanofiber Preparation

PVA solutions (6, 8, and 10 wt %) were prepared by dissolving known quantities of PVA in DI water. The mixtures were mechanically stirred (VELP ARE heating magnetic stirrer, VELP Scientifica, Usmate, Italy) in a flask for 60 min to fabricate emulsion PVA microbubbles. During the process, the parameters impacting PVA bubble forming were determined by varying the stirring rate and heating temperature. The stirring rates were set at 300, 700, and 1100 rpm, while the heating temperature was varied at 40, 60, and 80 °C. PVA solutions (without bubbles) were prepared (at 60 °C, 100 rpm for 60 min) to produce fibers through conventional ES.

**Figure 1:**
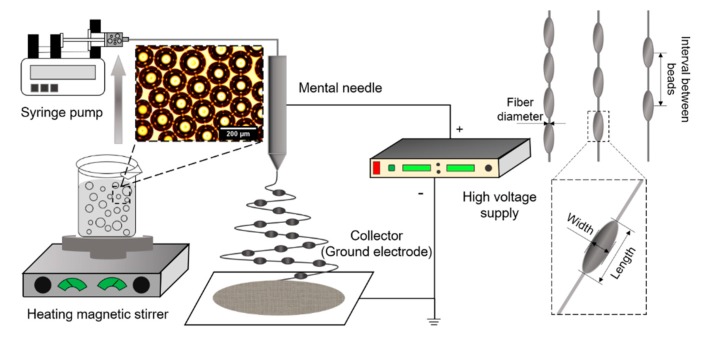
Schematic diagram of an electrospinning (ES) apparatus for beaded fiber engineering and graphical illustration of the key fiber properties assessed in this study.

The ES apparatus and techniques for uniform fiber preparation have been described in previous publications [19,20,21]. As shown in Figure 1, the ES apparatus consists of a high power voltage supply (Glassman high voltage Inc. High Bridge, series FC, NJ, USA), a high-precision programmable syringe pump (KDS100, KD Scientific, Holliston, MA, USA), and a stainless steel needle. Grounded aluminum foil was used as the fiber collector substrate, and was set 13 cm directly below the spinning needle. Individual PVA microbubble suspensions were loaded into 5-mL plastic syringes, and perfused from the syringe into the processing needle (the inner and outer diameters were 0.8 mm and 1 mm, respectively) via silicon tubing. The flow rate was maintained at 0.5 mL/h. During the ES process, an electric field, ranging between 14–18 kV, was applied between the needle and the ground electrode (round-shape electrode with inner and outer diameters of 2.7 and 3.5 cm) to produce beaded PVA nanofibers. All experiments were performed at ambient temperature (25 °C).

#### 2.2.2. Beaded Nanofiber Characterization

Optical (OM, BMC503, Phenix, Shangrao, China) and field emission scanning electron (SEM, ProX-Phenom, PhenomWorld, Eindhoven, The Netherlands) microscopy were used to assess the size distribution and surface morphology of PVA microbubbles and electrospun fibers. Prior to SEM analysis, samples were coated with a layer of gold under a vacuum environment for 60 s, and then observed at an accelerating voltage of 10 kV. Optical and electron micrographs were analyzed using ImageJ software (NIH, Bethesda, MD, USA) to obtain the bubble, bead, fiber, and AR of various structures. A sample size of 100 was selected for each condition. The AR of the beads was calculated using Equation (1):

(1) $$\mathrm{Aspect}\;\mathrm{ratio}\;(\mathrm{AR})=\frac{\mathrm{Width}\;\mathrm{of}\;\mathrm{the}\;\mathrm{bead}}{\mathrm{Length}\;\mathrm{of}\;\mathrm{the}\;\mathrm{bead}}\times 100\%$$

Water contact angle (CA) values of different electrospun fibers were measured using an optical contact angle meter (SL200KB, KINO Industry Co. Ltd., Norcross, GA, USA) to confirm the surface hydrophilicity of fabricated membranes. Fibrous membranes were prepared by collecting the electrospun PVA fibers on precisely located object slides for 15 min under constant ES conditions. One hour after the collection, the mean CA was determined by the average of left and right CAs of a single 1-μL water droplet pipetted on each membrane sample. CAs were recorded when the pipetted droplet was stable (2 s post droplet falling). The resulting value of each measurement represents the average value of 10 measurements. All statistical diagrams were plotted using Origin software (OriginLab, Northampton, MA, USA).

Fourier Transform Infrared (FTIR) spectroscopy was set to determine the chemical interactions and materials stability of electrospun PVA fibers and TE-HCL. For each measurement, 2 mg of sample (pure electrospun PVA fibers, TE-HCL, or the composite fibers) was dispersed in 200 mg KBr medium by grinding in a mortar. The mixture was then compressed into a transparent pellet at a pressure of 20 MPa. The fabricated pellet was scanned with FTIR (IR-Affinity1, Shimadzu, Kyoto, Japan), ranging from 3500 to 500 cm^−1^. Spectra were obtained using 20 scans per sample.

#### 2.2.3. In Vitro Drug Release Assessment

TE-HCL release from PVA matrices was studied to evaluate the effect of beads-on fiber morphology on the drug release profile. PVA/TE-HCL composite electrospun fibers with various morphologies (beads-on fibers, stretched beads-on fibers, and fibers without beads) were prepared using PVA solutions with different concentrations (6, 8, and 10 wt %), and the TE-HCL content in each solution was constantly set as 4 wt %. Then, 20 mg per electrospun sample was placed into 10 mL of PBS. At designated time intervals, 2 mL of supernatant was removed from the medium for measurement, and then the medium was compensated with fresh PBS to 10 mL. The concentration of TE-HCL in PBS was determined by measuring absorbance at 363 nm using a UV-2600 spectrophotometer (Shimadzu, Kyoto, Japan) [22]. The cumulative release of TE-HCL was determined using Equation (2) [23]:

(2) $$\mathrm{Cumulative}\text{ TE-HCL }\mathrm{release}\;(\mu g)=\sum {10C}_{t}-{8C}_{t-1}$$

where *C*_t_ is the concentration of TE-HCL in PBS medium at time *t*, and *C*_t−1_ is the value at the designed time point before *t*. All the experiments were performed in triplicate.

### 2.3. Statistical Analysis

All experiments were performed in triplicate and the data are presented as means ± standard deviation (*n* = 3). Statistical analysis was carried out using SPSS software (SPSS Statistics v18, IBM, Chicago, IL, USA). Statistically significant differences between variables were assessed via one-way analysis of variance (ANOVA) followed by Student’s *t*-test (* *p* < 0.05).

## 3. Results and Discussion

For ES, the formation of uniform polymeric fibers is attributed to the stable jetting of a solution (or a liquid phase medium) under the influence of surface tension and an applied electrical potential [3]. In this instance, a microbubble suspension was selected as the process medium, with subsequent morphing of porous thin-film spherical structures to beaded fibers through electrically driven stretching.

**Figure 2:**
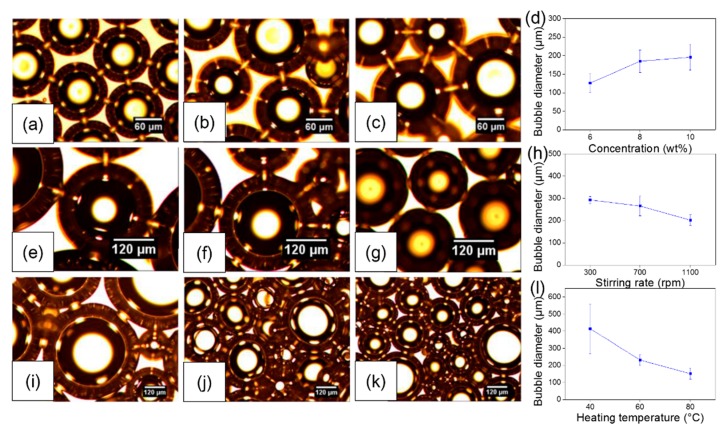
Effect of bubble preparation parameters on their size distribution. Optical micrographs of bubbles prepared using different PVA solution concentrations with all other parameters constant (stirring rate: 700 rpm; heating temperature: 60 °C): (**a**) 6; (**b**) 8; and (**c**) 10 wt %. (**d**) Bubble size distribution as a function of solution concentration. Optical micrographs of bubbles fabricated using various stirring rates with all other parameters constant (solution concentration: 10 wt %; heating temperature 60 °C): (**e**) 300; (**f**) 700; and (**g**) 1100 rpm. (**h**) Bubble size distribution as a function of stirring rate. Optical micrographs of bubbles produced at different heating temperatures with all other parameters constant (solution concentration: 10 wt %; stirring rate: 700 rpm): (**i**) 40; (**j**) 60; and (**k**) 80 °C. (**l**) Bubble size distribution as a function of solution temperature.

PVA microbubbles were prepared using a high shear emulsification process. For this, the PVA solution concentration, stirring rate, and heating temperature were systematically optimized. Figure 2a–c show optical micrographs of PVA microbubbles prepared using 6, 8, and 10 wt % PVA solutions, respectively, with a fixed stirring rate and heating temperature of 700 rpm and 60 °C, respectively. PVA microbubbles were generated for all polymer concentrations. Increasing the solution PVA concentration yielded coarser microbubbles, as shown in Figure 2d. Mean bubble diameters were 126.2 ± 24.8, 185.0 ± 30.7, and 196.0 ± 34.7 μm when prepared using 6, 8, and 10 wt % PVA solutions, respectively. The increase in bubble diameter is attributed to the increasing PVA solution viscosity, which correlates positively with the PVA concentration [24,25]. Low solution concentrations facilitate the mixing of air and PVA solutions through a finer surface film coat integrity. Furthermore, during the stirring process, coarser bubbles prepared from low polymer concentrations break into smaller daughter bubbles which are thermodynamically more stable. Thus, compared to bubbles produced using 8 and 10 wt % PVA solutions, bubbles from 6 wt % PVA solution are smaller in size and more uniform.

The effect of the stirring rate on PVA micro-bubble formation and size was investigated by systematically varying the parameter in the range of 300–1100 rpm. The solution concentration and temperature were constant and set to 10 wt % and 60 °C, respectively. Figure 2e–g show optical micrographs of PVA microbubbles prepared at stirring rates of 300, 700, and 1100 rpm, respectively. PVA microbubbles remain spherical under these conditions and their mean diameters decreased with increasing stirring rates, as shown in Figure 2h. Mean PVA bubble diameters decreased from 292.6 ± 16.1 μm at 300 rpm to 202.0 ± 23.7 μm at 1100 rpm. At high stirring rates, coarse bubbles in the solution burst into daughter bubbles due to intense shear force, resulting in smaller and more stable bubbles [12,26].

Environmental temperature is also a significant parameter in bubble formation, as elevated temperatures during synthesis decrease the viscosity of a polymeric solution. The effect of temperature on PVA microbubble diameter was investigated by manipulating the heating temperature (40, 60, and 80 °C) during the stirring process, while all other parameters were constant (concentration: 10 wt % and stirring rate: 700 rpm). Figure 2i–k shows PVA microbubbles prepared at 40, 60, and 80 °C, possessing mean diameters of 414.2 ± 146.1, 231.5 ± 32.2, and 151.6 ± 33.4 μm, respectively (Figure 2l). Microbubbles remain spherical, although a reduction in mean diameter is observed as a function of stirring temperature, which can be attributed to increased diffusion and leakage of gas from within the encapsulating thin polymeric film coat.

**Figure 3:**
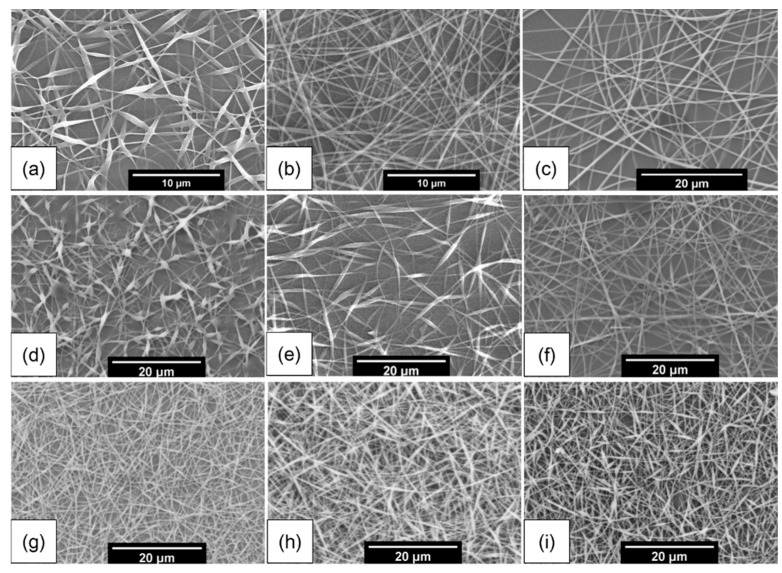
Scanning electron micrographs. Nanofibers produced using various pure PVA solutions (parameters 60 °C, 100 rpm for 60 min; all other conditions are constant): (**a**) 6; (**b**) 8; and (**c**) 10 wt %. Nanofibers produced using various PVA microbubble suspensions (all other parameters constant): (**d**) 6; (**e**) 8; and (**f**) 10 wt %. Nanofibers prepared using PVA microbubble suspensions and various applied voltages (all other parameters constant): (**g**) 14; (**h**) 16; and (**i**) 18 kV.

The polymer concentration influences both the viscosity and surface tension of an ES solution [27]. In this study, PVA liquids containing no microbubbles (prepared at 100 rpm, PVA solutions at 6, 8, and 10 wt %) and microbubble suspensions (prepared at 700 rpm using 6, 8, and 10 wt % PVA) were used to synthesize beaded fibers. All other parameters were constant (flow rate: 0.5 mL/h; applied voltage: 16 kV; deposition distance: 13 cm). Figure 3a–c indicate that nanofiber production using neat PVA solutions (without microbubbles) give rise to varying microstructures. Beaded fibers were formed with 6 wt % PVA solution only, while uniform continuous structures were produced when 8 and 10 wt % PVA solutions were used. However, when microbubble suspensions were used, both 6 and 8 wt % PVA suspensions yielded beaded fibers, which were more pronounced as shown in Figure 3d,e. Increasing the suspension concentration to 10 wt % led to pristine PVA fiber production (Figure 3f). Compared to neat PVA solution deployment, the utilization of a microbubble suspension facilitates beaded nanofiber production at a greater polymer concentration (8 wt %), which is valuable when increased polymer content for fabricated structures is essential (e.g., for structural integrity or controlled release via enhanced matrix-based diffusion). Furthermore, microbubble ES has also been shown to increase throughput rates which make the process attractive from a production rate perspective. The result indicates a potential forming route for beaded fibers using relatively high polymer concentrations through the substitution of neat polymeric solutions with a respective polymeric microbubble suspension. The microbubble coat is a flexible polymeric film, (capable of) hosting gas within its core. As gas diffuses from the core, the polymeric coating remains intact and bubble shrinkage is observed. The interfacial tension between the gaseous-liquid phases keeps the coating intact and a greater density (per unit space) of polymer accumulates around the escaping gas, which has been observed and utilized in previous studies to prepare porous films and scaffolds [28,29]. This clearly suggests that the density of polymeric chains in the encapsulating film is much greater than a homogenously distributed pristine polymer solution. Therefore, at 6 and 8 wt %, when suspensions are elongated and stretched during EHDA, a critical difference between polymer content in microbubble coating thin film and media during process stretching exists. This manifests as beaded structures. Comparing 8 wt % bubble and pure PVA media, the former retains irregularity in polymer homogeneity through the bubble structure. At higher suspension concentrations (e.g., 10 wt %), it is speculated there is sufficient polymer chain merging, due to sufficiently increased polymer content, to enable chain entanglement during the EHDA jetting-elongation process. Overall, this is a valuable method where mechanical properties and structural integrity need to be enhanced through increasing polymer loading content.

Applied voltage is another crucial factor. Sustained fiber jetting occurs when the applied voltage exceeds the threshold ES voltage. The polymer concentration (for bubble suspensions) was fixed to 8 wt % and the applied voltage was increased from 14 to 18 kV. A variation in the resulting PVA fiber morphology is shown in Figure 3g–i. Increasing the applied voltage leads to pronounced beaded fiber morphology. This is explained due to an increase in the electrostatic repulsive force on the charged jet which is known to favor fiber narrowing, thus increasing the probability of bead formation [5,30].

**Figure 4:**
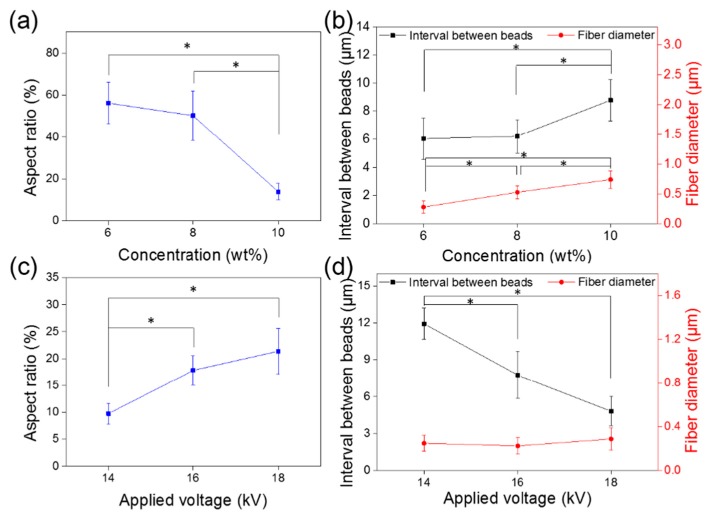
Effect of solution concentration on fiber properties: (**a**) bead AR; (**b**) inter-bead distance. Effect of applied voltage on fiber properties: (**c**) bead AR; (**d**) inter-bead distance. (* *p* < 0.05).

In order to evaluate the process parameters on the resulting beaded fiber morphology, AR, inter-bead distance, and diameter were measured. As shown in Figure 4a, increasing the PVA concentration from 6 to 10 wt % (for suspensions), leads to a reduction in fiber AR (56% to 14%). Fiber structure also deforms from near uniform to stretched spindle-format, which is attributed to the increased viscosity at high PVA concentrations [31]. The inter-bead distance and fiber diameter both appear to increase with the polymer concentration (Figure 4b). When the PVA bubble forming concentration is increased from 6 to 10 wt %, the inter-bead distance increases from 6.0 to 8.8 μm with the fiber diameter surging from 277.6 to 737.7 nm. This indicates that solution viscosity can be used to control bead density and spacing during the forming process.

The effect of voltage on beaded fiber morphology can be seen in Figure 4c,d. Greater applied voltage leads to an increase in the electrostatic repulsive force on the fluid jet, which leads to an unstable electrical process, and is a factor giving rise to beaded structures [27]. As the applied voltage was increased from 14 to 18 kV, the AR also increased from 10% to 21%, and the inter-bead distance decreased from 11.9 to 4.8 μm. There was no clear variation in fiber diameter (from 247.8 to 287.0 nm), although the standard deviation of synthesized fiber diameters at 18 kV was larger (±102.2 nm) than those prepared at 14 and 16 kV (±72.8 and ±73.0 nm, respectively). This further supports beaded fiber preference at elevated applied voltages.

**Figure 5:**
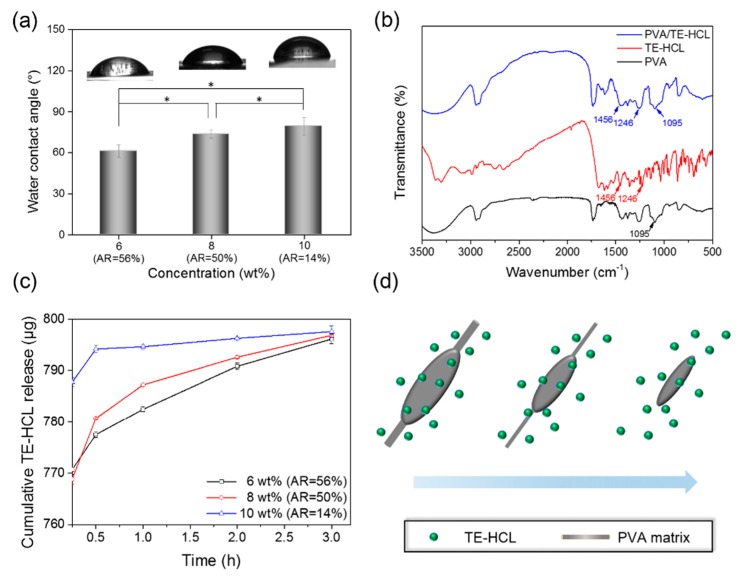
(**a**) Water contact angles on PVA fibrous membranes prepared using 6, 8, and 12 wt % PVA solutions (bead fiber AR = 14%, 50%, and 56%, respectively). (**b**) FTIR spectra of neat materials and samples prepared in this study. (**c**) TE-HCL release profiles from PVA matrices prepared using various solution concentrations. (**d**) Schematic illustration of drug release from PVA beaded fibers over time (* *p* < 0.05).

Biomaterial surface hydrophilicity is paramount for biological applications [32]. Figure 5a shows water contact angles (CAs) on selected beaded PVA membranes prepared using various PVA microbubble suspensions. CAs for 6, 8, and 10 wt % PVA membranes (with corresponding ARs: 56%, 50%, and 14%) are 61.2 ± 4.6°, 73.8 ± 2.9°, and 79.4 ± 6.4°, respectively. These results indicate that all fibrous PVA membranes are hydrophilic, which is in accordance with a previous report featuring this polymer [33]. The variation in PVA membrane hydrophilicity is attributed to surface morphology, and this is directly linked to the PVA concentration in the processing medium. As shown in Figure 3d–f, fibrous mats with/without beads exhibit discontinuous surface topographies. Compared to uniform fiber PVA membranes, beaded mats exhibit a coarser surface and reduced air entrapment at the interface, which facilitates surface contact [32,34].

In order to investigate potential active release applications of beaded fibers, TE-HCL drug was encapsulated into fibrous PVA matrices during synthesis. FTIR spectroscopy confirmed the presence of PVA and encapsulated TE-HCL within fibrous mats (Figure 5b). A characteristic absorption signal of PVA, indicating C=O stretching, was observed at 1095 cm^−1^ [35]. The main bands for TE-HCL were found at 1246 and 1456 cm^−1^, indicating C–C stretching and O–H bending, respectively [22]. FTIR analysis demonstrated the successful encapsulation of TE-HCL into PVA fibers.

TE-HCL release (Figure 5c) from various matrices indicates modulated release behavior from beaded structures. TE-HCL release from fibrous membrane synthesized using 10 wt % PVA solution (bead fiber AR = 14%) is much quicker than matrices prepared using 6 and 8 wt % (beaded fiber ARs = 56 and 50, respectively). The release of TE-HCL from fibrous mats prepared with 10 wt % PVA solution increased to 794.2 μg after 0.5 h of in vitro assessment, and continued steadily to 797.6 μg at the 3-h time interval. On the other hand, for fibrous membranes formed with 6 and 8 wt % PVA bubble suspensions, the cumulative release quantities of TE-HCL were 777.5 and 780.7 μg, respectively, at 0.5 h. At the 3-h time interval, TE-HCL quantities (released) reached 796.1 and 796.9 μg, respectively. The difference in released quantities is attributed to spatial TE-HCL distribution within respective PVA matrices. As Figure 5d suggests, compared to non-beaded PVA structures, TE-HCL encapsulated within ‘beads’ provide a combinatorial effect of active release from two structures; particle and fiber [11,12]. However, the current study provides a highly valuable approach for the design and fabrication of beaded nanofibers, which have increasing potential in drug delivery and biomedical engineering [9,13]. Further explorations will focus on exploiting this new approach to fabricate multi-segment and composite fibers for on-demand active release strategies.

## 4. Conclusions

In summary, beaded fibers with mean diameters ranging from 223 to 737 nm and ARs ranging from 10% to 56% were fabricated using PVA microbubble suspensions. Bead formation and size distribution were regulated via PVA solution concentration and applied voltage. Greater PVA concentration deployment yielded fibrous membranes with superior hydrophobic properties. In addition, FTIR analysis indicates the successful and stable encapsulation of TE-HCL within the PVA fiber matrix. Drug release from beaded structures and membrane hydrophilicity are influenced by their respective ARs. These findings indicate a method to tailor fibrous beaded structures for biomedical and drug delivery applications.
